# Designing, synthesis and characterization of 2-aminothiazole-4-carboxylate Schiff bases; antimicrobial evaluation against multidrug resistant strains and molecular docking

**DOI:** 10.1186/s13065-019-0631-6

**Published:** 2019-09-14

**Authors:** Saima Ejaz, Humaira Nadeem, Rehan Zafar Paracha, Sadia Sarwar, Sadaf Ejaz

**Affiliations:** 1 Department of Pharmaceutical Chemistry, Riphah Institute of Pharmaceutical Sciences, Riphah International University, Islamabad, Pakistan; 2 Research Center for Modeling and Simulation, National University of Science and Technology, Islamabad, Pakistan; 3 Department of Pharmacognosy, Riphah Institute of Pharmaceutical Sciences, Riphah International University, Islamabad, Pakistan; 4 Microbiology and Public Health Laboratory, Department of Biosciences, COMSATS University Islamabad, Islamabad, Pakistan

**Keywords:** 2-Aminothiazole, Antimicrobial evaluation, Minimum inhibitory concentration, Antifungal activity, Molecular docking, Schiff bases

## Abstract

**Background:**

2-Aminothiazoles are significant class of organic medicinal compounds utilized as starting material for the synthesis of diverse range of heterocyclic analogues with promising therapeutic roles as antibacterial, antifungal, anti-HIV, antioxidant, antitumor, anthelmintic, anti-inflammatory & analgesic agents.

**Experimental:**

Eight compounds 1a, 2a–2g were synthesized and characterized by FTIR and NMR (^1^H and ^13^C). Evaluation of antibacterial potential against multi-drug resistant clinical isolates was performed and minimum inhibitory concentration (MIC) values were determined. Antifungal activity was also performed. Protein–ligand interactions of compounds with target enzyme were evaluated through docking studies.

**Results:**

Resistance profiling of bacterical clinical isolates (MDRs) depicted that some standard drugs used were not active against these MDRs while our synthesized compounds showed good MIC values. Among all the synthesized compounds, 2a and 2b showed significant antibacterial potential towards gram-positive *Staphylococcus epidermidis* and gram-negative *Pseudomonas aeruginosa* at MIC 250 µg/mL and 375 µg/mL respectively. Likewise, compound 2d and 2g exhibited inhibitory potential against gram-positive *Staphylococcus aureus* and gram-negative *Escherichia coli* at MIC values of 250 and 375 µg/mL respectively. Compound 2b showed maximum antifungal potential against *Candida glabrata* (ATCC 62934) with a zone of inhibition 21.0 mm as compared to the reference drug nystatin which showed lesser antifungal potential with a zone of inhibition of 19.1 mm. *Candida albicans* (ATCC 60387) showed maximum sensitivity to compound 2a with a zone of inhibition 20.0 mm. Its antifungal activity is more in comparison to reference drug nystatin with exhibited the zone of inhibition of 19.3 mm. Designed compounds were docked with the target enzyme UDP-*N*-acetylmuramate/l-alanine ligase. The compound 2b showed highest binding affinity (− 7.6 kcal/mol).

**Conclusions:**

The synthesized compounds showed moderate to significant antibacterial and antifungal potential. It is clear from the binding affinities that compounds having hydroxyl group substituted on benzene ring possess strong binding affinity as compared to other analogues. These designed compounds could be considered to act as antagonists against target UDP-*N*-acetylmuramate/l-alanine ligase.

## Introduction

Schiff bases have gained remarkable place in medicinal chemistry because of their diverse therapeutic roles as antibacterial [1, 2], antifungal [3–6], anti-HIV [7], antioxidant, antitumor, anthelmintic [8], anti-inflammatory & analgesic agents [9]. Thiazole nucleus is present in both natural and synthetic products with notable pharmacological and therapeutic activities [10]. Thiamin also called as vitamin B1, also contains thiazole nucleus. Thiamin functions in human body as co-enzyme in metabolic pathways of carbohydrates and amino acids [11]. Synthesis of 2-amino-6-methylbenzothiazoles Schiff bases with antibacterial activity comparable to that of ampicillin, are reported [12]. 2-amino-4-substituted thiazoles are already reported for their anthelmintic, anti-leukotrienes, anticonvulsant, antimalarial and fungicidal properties [13].

The registered drugs Mirabegron (Anticholinergic agent) and Cefdinir (Antibacterial agent) belong to aminothiazole analogues [14, 15]. Since it is well known that 2-aminothiazoles are significant class of organic medicinal compounds utilized as starting material for the synthesis of diverse range of heterocyclic analogues. Therefore, the design and synthesis of ethyl-2-aminothiazole-4-carboxylate Schiff bases was targeted in this study.

Designing of evident drugs can be achieved through virtual screening and molecular docking strategies. Likewise, interpretation of probable drug specificity with target enzyme or protein can be assessed [16]. In the present study the selected uridine diphosphate-*N*-acetylmuramate/l-alanine ligase was selected as antimicrobial target for docking [17]. It is the bacterial enzyme that catalyzes the reaction of peptidoglycan synthesis by first amino acid addition to peptidoglycan sugar moiety. Peptidoglycan is the key element for bacterial cell wall [18]. By targeting this ligase enzyme, the bacterial cell integrity can be dismantled ultimately resulting in bacterial cell death.

## Experimental

### Molecular docking

#### Ligand designing

**Fig. 1:**
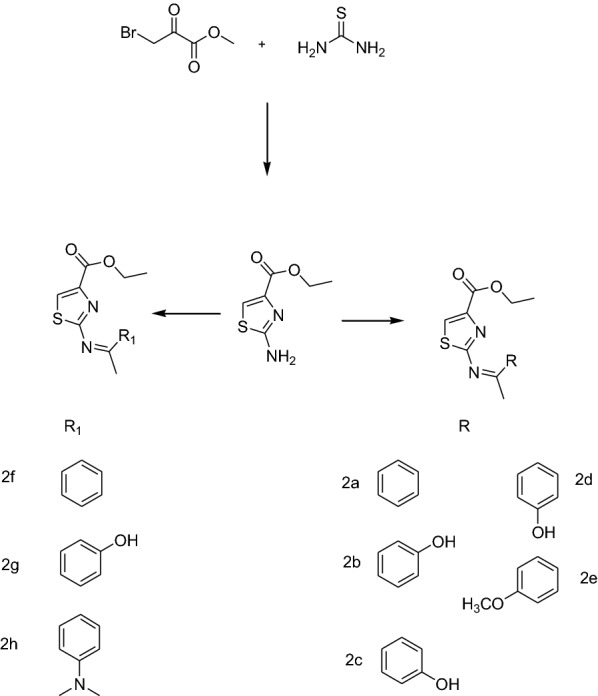
General scheme for the synthesis of ethyl 2-aminothiazole-4-carboxylate and its derivatives

Eight ligands were designed using ChemSketch 12.0 (https://www.acdlabs.com/resources/freeware/). Analogues of ethyl -2-aminothiazole-4-carboxylate were designed by altering the amino group as illustrated in Fig. 1. Four aldehydes and two ketones were used to design the compounds form 2a–2h. Molecular properties were determined using ChemSketch Tool (https://www.acdlabs.com/resources/freeware/). Chemo-informatics, ADME properties and drug likeliness were determined by following Lipinski rule while associated parameters of designed ligands were predicted by Molinspiration (https://www.molinspiration.com/) and pkCSM online tool [19, 20]. The toxicity profile was obtained using TOXTREE and pkCSM online tool [21].

#### Pocket identification and docking of designed compounds

Pocket identification was accomplished via Dogsitescorer [22]. Crystal structure of bacterial target enzyme UDP-*N*-acetylmuramate/l-alanine ligase was taken from databank (https://www.rcsb.org/) (PDB ID: 1GQQ). Enzyme-Ligand docking was accomplished with PyRx [23], via AutoDock VINA module [24]. Docking coordinates of x = 42.396, y = 47.393 and z = 84.654 with dimensions x = 47.187, y = 23.629 and z = 38.776 were used according to the best binding site predicted by DogSiteScorer. Open Babel GUI and Discovery Studio 2017 R2 Client (https://www.3dsbiovia.com/) were utilized for preliminary procedures and elaboration of enzyme ligand interactions respectively [25].

### Chemistry

Ethyl bromopyruvate (technical grade, 90%), thiourea (ACS reagent) benzaldehyde (ReagentPlus^®^ 99%), salicylaldehyde (reagent grade, 98%), 3-hydroxybenzaldehyde (AR ≥ 99%), 4-hydroxybenzaldehyde (Analytical standard), 4-hydroxy-3-methoxybenzaldehyde (ReagentPlus^®^ 99%), acetophenone (Analytical standard), 2′-hydroxyacetophenone (ReagentPlus^®^ 99%),. Ethanol (absolute, ACS reagent) and glacial acetic acid (100%, anhydrous for analysis ACS, ISO reagent) were used purchased from Sigma Aldrich and Merck. Synthesized compounds were purified by recrystallization in appropriate solvents and examined through thin layer chromatography (Merck Silica gel 60 F_254_). Melting points were determined by using digital Gallenkamp model MPD BM 3.5 apparatus. Characterization of synthesized compounds was made through spectrophotometric analysis; FT-IR (Thermoscientific NICOLET IS10 spectrophotometer), ^1^H & ^13^C NMR (Bruker AM-300 and AM-100 spectrophotometer) using DMSO and CDCl_3_ respectively. Elemental analysis values were recorded on Model ANALYST 2000 CHNS, Perkin Elmer Analyzer. Multiskan™ GO Microplate Spectrophotometer was used to quantify synthesized compounds for accuracy, precision and sensitivity [26].

#### General procedure for the preparation of ethyl 2-aminothiazole-4-carboxylate (**1a**)

Ethyl bromopyruvate (2 mol) and of thiourea (3 mol) in 100 mL of ethanol (99.9%) was refluxed for 24 h. Progress of the reaction was monitored by TLC (petroleum ether: ethyl acetate, 1:3). After completion, the reaction mixture was cooled to room temperature, concentrated and poured into ice cold water and basified (pH 10) with 2 M NaOH resulting in separation of off-white precipitation that was recrystallized using ethanol.

Yield 70%, m.p. 175–177 °C, R_f_ 0.71 (petroleum ether: ethyl acetate, 1:3); IR (KBr) cm^−1^: 1690 (C=O ester), 3000 (C–H), 3300–3150 (N–H amine), 1566 (C=C); ^1^H-NMR (Cholroform, δ ppm): δ = 4.34 (q, 2H, CH_2_), 1.38 (t, 3H, CH_3_), 7.41 (s, 1H, Thiazole), 5.85 (s, 2H, NH_2_); Elemental analysis: C_6_H_8_N_2_O_2_S, Calculated: C 41.81%, H 4.65%, N 16.26% O 18.59% S 18.59%; Found C 41.79%, H 4.64%, N 16.26% O 18.58% S 18.57%.

#### General procedure for the synthesis of ethyl 2-aminothiazole-4-carboxylate derivatives

Ethyl 2-aminothiazole-4-carboxylate (0.05 mol) and aldehyde/ketone (0.05 mol) in absolute ethanol (30 mL) were dissolved and few drops of glacial acetic acid were added. Reaction mixture was stirred and refluxed for 12 h. Progress of reaction was monitored by TLC. After cooling the excess solvent was evaporated by using rotary evaporator. The residue was dissolved in ethyl acetate, crystal growth was observed after few days [27, 28]. Physical data of the synthesized compounds is given in Table 1.

**Table 1:** Physical data of synthesized compounds (**1a**, **2a**–**2g**)

Comp	Molecular formula	MW calculated (g/mol)	M.P (°C)	Physical state	Color	% Yield	R_f_ Value
**1a**	C_6_H_8_N_2_O_2_S	172.20	175–177	Solid	Off white-pale yellow	70	0.71
**2a**	C_13_H_12_N_2_O_2_S	260.31	160–162	Crystal	Light brown	50	0.61
**2b**	C_13_H_12_N_2_O_3_S	276.31	198–199	Solid	Light brown	56	0.64
**2c**	C_13_H_12_N_2_O_3_S	276.31	195–197	Solid	Orange	54	0.66
**2d**	C_13_H_12_N_2_O_3_S	276.31	200–202	Solid	Brown	60	0.69
**2e**	C_14_H_14_N_2_O_4_S	306.33	196–198	Solid	Light brown	50	0.61
**2f**	C_14_H_14_N_2_O_2_S	274.33	190–192	Crystal	Light brown	52	0.59
**2g**	C_14_H_14_N_2_O_3_S	290.33	164–165	Crystal	Light brown	62	0.60

Solvent system: ethyl acetate: petroleum ether (3:1), TLC silica HF-254

##### Ethyl 2-{[(*E*)-phenylmethylidene]amino}-1,3-thiazole-4-carboxylate (2a)

Yield 50%, m.p. 160–162 °C, R_f_ 0.61 (petroleum ether: ethyl acetate, 1:3); IR (KBr) cm^−1^: 1687 (C=O ester), 3023 (C–H), 1513 (C=C), 1616 (C=N);^1^H NMR (DMSO, δ ppm): δ = 4.25 (q 2H CH_2_), 1.26 (t, 3H, CH_3_), 7.44 (s, 1H, Thiazole), 7.02 (m, 5H, Ar), 9.47 (s, 1H, H–C=N); 13C NMR (CDCl3, d ppm): 166.3 (C5), 165.2 (C12), 162.2 (C7), 143.7 (C2), 134.7 (C13), 130.7 (C18), 129.5 (C14), 129.5 (C15), 127.8 (C16), 127.8 (C17), 122.5 (C1), 61.3 (C10), 13.8 (C11); Elemental analysis: C_13_H_12_N_2_O_2_S, Calculated: C 59.93%, H 4.61%, N 10.76%; Found C 59.91%, H 4.60%, N 10.76%.

##### Ethyl 2-{[(*E*)-(2-hydroxyphenyl)methylidene]amino}-1,3-thiazole-4-carboxylate (2b)

Yield 56%, m.p. 198–199 °C, R_f_ 0.64 (petroleum ether: ethyl acetate, 1:3); IR (KBr) cm^−1^: 1687 (C=O ester), 2975 (C–H), 1535 (C=C), 1617 (C=N), 3261 (OH); ^1^H NMR (DMSO, δ ppm): δ = 4.20 (q, 2H, CH_2_), 1.24 (t, 3H, CH_3_), 7.45 (s,1H, Thiazole), 7.40 (m, 4H, Ar), 9.76 (s,1H, H–C=N), 7.26 (s, 1H, Ar–OH); 13C NMR (CDCl3, d ppm): 165.1 (C5), 164.1 (C12), 162.9 (C7), 161.1 (C14), 143.1 (C2), 133.7 (C18), 133.5 (C15), 122.8 (C1), 120.8 (C17), 117.8 (C13), 116.9 (C16), 61.1 (C10), 14.4 (C11); elemental analysis: C_13_H_12_N_2_O_3_S, calculated: C 56.458%, H 4.343%, N 11.58%; found C 56.45%, H 4.34%, N 11.58%.

##### Ethyl 2-{[(*E*)-(3-hydroxyphenyl)methylidene]amino}-1,3-thiazole-4-carboxylate (2c)

Yield 54%, m.p. 195–197 °C, R_f_ 0.66 (petroleum ether: ethyl acetate, 1:3); IR (KBr) cm^−1^ 1690 (C=O ester), 2983 (C–H), 1502 (C=C), 1614 (C=N), 3259 (OH); ^1^H NMR (DMSO, δ ppm): δ = 4.21 (q, 2H, CH_2_), 1.24 (t, 3H, CH_3_), 7.43 (s, 1H, Thiazole), 7.30 (m, 4H, Ar), 8.89 (s,1H, H–C=N); 13C NMR (CDCl3, d ppm): 166.8 (C5), 164.9 (C12), 161.9 (C7), 158.9 (C16), 142.4 (C2), 135.7 (C13), 129.5 (C17), 121.9 (C15), 121.3 (C1), 116.3 (C18), 115.6 (C14), 61.9 (C10), 14.1 (C11); elemental analysis: C_13_H_12_N_2_O_3_S, calculated: C 56.458%, H 4.343%, N 11.58%; found C 56.45%, H 4.34%, N 11.57%.

##### Ethyl 2-{[(*E*)-(4-hydroxyphenyl)methylidene]amino}-1,3-thiazole-4-carboxylate (2d)

Yield 60%, m.p. 200–202 °C, R_f_ 0.69 (petroleum ether: ethyl acetate, 1:3); IR (KBr) cm^−1^: 1688 (C=O ester), 2953 (C–H), 1517 (C=C), 1612 (C=N), 3250 (OH); ^1^H NMR (DMSO, δ ppm): δ = 4.25 (q, 2H, CH_2_), 1.25 (t, 3H, CH_3_), 7.42 (s, 1H, Thiazole), 7.20 (m, 4H, Ar), 9.48 (s, 1H, H–C=N); 13C NMR (CDCl3, d ppm): 167.1 (C5), 163.8 (C12), 161.9 (C7), 157.3 (C18), 142.4 (C2), 131.5 (C14), 131.5 (C15), 127.7 (C13), 121.2 (C1), 117.8 (C16), 117.8 (C17), 61.6 (C10), 14.2 (C11); elemental analysis: C_13_H_12_N_2_O_3_S, calculated: C 56.458%, H 4.343%, N 11.58%; found C 56.45%, H 4.33%, N 11.56%.

##### Ethyl 2-{[(*E*)-(4-hydroxy-3-methoxyphenyl)methylidene]amino}-1,3-thiazole-4-carboxylate (2e)

Yield 50%, m.p. 196–198 °C, R_f_ 0.61 (petroleum ether: ethyl acetate, 1:3); IR (KBr) cm^−1^: 1696 (C=O ester), 3030 (C–H), 1506 (C=C), 1619 (C=N), 3210 (OH); ^1^H NMR (DMSO, δ ppm): δ = 4.22 (q, 2H, CH_2_),1.26 (t, 3H, CH_3_), 7.45 (s, 1H, Thiazole), 7.01 (m, 3H, Ar), 8.40 (s, 1H, H–C=N), 3.37 (s, 3H, OCH_3_), 7.25 (s, 1H, Ar–OH); 13C NMR (CDCl3, d ppm): 165.9 (C5), 165.1 (C12), 162.9 (C7), 147.8 (C16), 146.2 (C18), 142.7 (C2), 125.7 (C13), 124.2 (C15), 121.2 (C1), 115.1 (C17), 112.8 (C14), 61.9 (C10), 57.5 (C21), 14.2 (C11); Elemental analysis: C_14_H_14_N_2_O_4_S, Calculated: C 54.842%, H 4.57%, N 9.14%; Found C 54.84%, H 4.57%, N 9.14%.

##### Ethyl 2-{[(1*E*)-1-phenylethylidene]amino}-1,3-thiazole-4-carboxylate (2f)

Yield 52%, m.p. 190–192 °C, R_f_ 0.59 (petroleum ether: ethyl acetate, 1:3); IR (KBr) cm^−1^: 1690 (C=O ester), 2985 (C–H), 1553 (C=C), 1617 (C=N); ^1^H NMR (DMSO, δ ppm): δ = 4.25 (q, 2H, CH_2_), 1.26 (t, 3H, CH_3_), 7.64 (s, 1H, Thiazole), 7.14 (m, 5H, Ar), 3.38 (s, 3H, CH_3_–C=N); 13C NMR (CDCl3, d ppm): 175.1 (C12), 168.8 (C5), 162.2 (C7), 142.7 (C2), 136.5 (C13), 131.7 (C18), 129.1 (C16), 129.1 (C17), 128.5 (C14), 128.5 (C15), 124.1 (C1), 61.9 (C10), 18.2 (C19), 14.3 (C11); elemental analysis: C_14_H_14_N_2_O_2_S, calculated: C 61.24%, H 5.103%, N 10.206%; found C 61.24%, H 5.10%, N 10.21%.

##### Ethyl 2-{[(1*E*)-1-(2-hydroxyphenyl)ethylidene]amino}-1,3-thiazole-4-carboxylate (2g)

Yield 62%, m.p. 164–165 °C, R_f_ 0.60 (petroleum ether: ethyl acetate, 1:3); IR (KBr) cm^−1^: 1685 (C=O ester), 2990 (C–H), 1570 (C=C), 1615 (C=N), 3437 (OH); ^1^H NMR (DMSO, δ ppm): δ = 4.21 (q, 2H, CH_2_), 1.23 (t, 3H, CH_3_), 7.45 (s, 1H, Thiazole), 7.25 (m, 4H, Ar), 3.34 (s, 3H, CH_3_–C=N); 13C NMR (CDCl3, d ppm): 178.9 (C12), 168.1 (C5), 160.9 (C7), 158.7 (C14), 141.7 (C2), 133.7 (C18), 129.9 (C15), 124.1 (C1), 123.7 (C13), 117.8 (C17), 114.2 (C16), 61.1 (C10), 19.5 (C19), 14.9 (C11); elemental analysis: C_14_H_14_N_2_O_3_S, calculated: C 57.89%, H 4.82%, N 9.65%; found C 57.87%, H 4.82%, N 9.64%.

### Antimicrobial assay

#### Resistance profiling

Analysis of resistance pattern of pathogenic microbe (resistant, intermediate, susceptible), antibiotic sensitivity assay was performed. Fresh cultures of bacterial strains were made on nutrient agar and incubated for 24 h at 37 °C. The following day isolated colonies from bacterial cultures were picked and dissolved in 1 mL of PBS (autoclaved normal saline) and inoculum turbidity was then confirmed with 0.5% McFarland standard [29].

Dried Muller Hinton (MH) agar plates were used for resistance profiling. With the help of sterilized syringe antibiotic discs of known concentration were placed on MH agar plates namely Cefepime, Ciprofloxacin, Imipenem, Cefoxitin, Ampicillin, Aztreonam, Tetracycline, Ceftazidime, Minocyclin, Gentamycin, Co-trimoxazole, Colistin, Clindamycin, Vancomycin, Doxycycline, Erythromycin and Chloramphenicol. Later these plates were incubated for 24 h at 37 °C. Afterwards, resistance pattern of particular strain was studied based upon the zone of inhibition measurement following the CLSI 2017 guideline [30, 31].

#### Quantification by nanophotometer

For quantification purpose Multiskan™ GO Microplate Spectrophotometer was used. The standard stock solution of 2 mg/mL was prepared for each active compound by adding 0.02 g in 10 mL distilled water. From this stock solution various working dilutions ranging from 40 to 2000 μg/mL were prepared in triplicate. Wave scan of the dilutions was performed between 200–900 nm and λ_max_ value was obtained from absorbance spectra. Afterwards, particular active compound λ_max_ was used to measure optical density of dilutions and standard curve was plotted.

#### Antibacterial activity and minimum inhibitory concentration

Antimicrobial activity was assessed by broth dilution method. Clinical isolates were obtained from Microbiology and Public Health Laboratory culture collection, COMSATS University, Islamabad. *Staphylococcus epidermidis* (MDR) and *Staphylococcus aureus* (MDR) were the gram-positive and *Escherichia coli* (MDR) and *Pseudomonas aeruginosa* (MDR) were the gram-negative strains selected for this study.

Broth dilution method was performed for antibacterial activity of synthesized compounds against selected clinical microbes. MIC was calculated by broth dilution method, stock solution of 2 mg/mL for each active compound with DMSO 20% as solvent was prepared which was further consumed for preparing working dilutions ranged from 40 to 1000 μg/mL. The selected model bacterial strains were subjected to these active compounds dilutions separately and OD values were obtained at 595 nm [32].

### Antifungal assay

Agar well diffusion method was utilized for antifungal assay using nystatin as positive control while DMSO (20%) was employed as a negative control. Sabouraud dextrose agar (SDA) and nutrient broth were prepared and sterilized by autoclaving at 121 °C for 15 min. Sterile agar plates were prepared by pouring the sterile agar in disposable sterile plates and incubated for 24 h for sterility check at 28 °C after congealing. The fungal strains *Candida albicans* (ATCC 60387) and *Candida glabrata* (ATCC 62934) were refreshed by inoculation in nutrient broth followed by 24 h incubation at 28 °C. Lawn of both fungal strains were made on nutrient agar plates and wells were made by employing sterile borer (6 mm). 50 μL of the synthesized compounds, nystatin and DMSO (20%) were poured through micropipette into individual well. The concentration of synthesized compounds, positives and negative controls used were 10 mg/mL and 750 μg/mL respectively. Plates were sealed by using paraffin film (Parafilm M) and incubated for 3–7 days at 28 °C to be examined for zone of inhibition that reflects the antifungal potential [33].

## Result and discussion

### Chemistry

Ethyl 2-aminothiazole-4-carboxylate was synthesized by reacting ethyl bromopyruvate and thiourea. Ethyl 2-aminothiazole-4-carboxylate (1a) was collected as off-white precipitates. Schiff bases 2a–2g were synthesized by reacting Ethyl 2-aminothiazole-4-carboxylate (1a) with different aldehydes and ketones as shown in Fig. 1.

Purity of all the synthesized compounds was ensured by recrystallization in appropriate solvents and checked by thin layer chromatography plates using ethyl acetate: petroleum ether (3:1) solvent system. Single spot yielded by each synthesized compound was obtained.

Synthesis of compound 1a was confirmed through FTIR and ^1^H NMR spectral data. Strong peak of C=O (ester) at 1690 cm^−1^, NH_2_ (amine) 3300–3150 cm^−1^ and C–H 3000 cm^−1^ stretch was observed in FTIR spectrum. ^1^H NMR spectrum showed singlet of amine and thiazole proton at 5.85 ppm and 7.41 ppm. Moreover, quartet and triplet of CH_2_ and CH_3_ was observed 4.34 ppm and 1.38 ppm respectively.

Confirmation of synthesized compounds was done by FTIR and ^1^H NMR spectral data. FTIR spectral data showed strong peak of C=O (ester) in case of each compound from 2a to 2e at cm^−1^: 1687, 1687, 1690, 1688 and 1696 respectively. Absence of amine peak and appearance of C=N (imine) confirmed the synthesis of Schiff bases in case of each derivative. FTIR spectral data showed strong peak of C=N (imine) in case of each compound from 2a to 2e at cm^−1^: 1616, 1617, 1614, 1612 and 1619 respectively. C–H stretch was observed at cm^−1^: 3023, 2975, 2983, 2953 and 3030 for compounds 2a–2e respectively. ^1^H NMR spectral data showed singlet of imine in case of each synthesized compound from 2a to 2e; ppm: 9.47, 9.76, 8.89, 9.48 and 8.42 respectively. Moreover, quartet of CH_2_ of synthesized compounds from 2a to 2e was observed a ppm: 4.25, 4.20, 4.21, 4.25 and 4.20 respectively. While triplet of CH_3_ in ^1^H NMR spectrum was observed in case of each compound from 2a to 2e at ppm: 1.26, 1.24, 1.24, 1.25 and 1.24 respectively.

Strong peak of C=O (ester) 1690 cm^−1^, C=N (imine) 1617 cm^−1^ and C–H stretch 2985 cm^−1^ were observed in FTIR spectrum of compound 2f. Singlets of CH_3_–C=N imine at 3.38 ppm and thiazole proton at 7.64 ppm were observed. Moreover, quartet and triplet of CH_2_ and CH_3_ were observed at 4.25 ppm and 1.26 ppm respectively. While in compound 2g peak of C=O (ester) was observed at 1685 cm^−1^. C=N (imine) 1615 cm^−1^ and C–H stretch 2990 cm^−1^ were also perceived in FTIR spectrum of compound 2g. ^1^H NMR spectral data showed singlets of CH_3_–C=N imine at 3.34 ppm and thiazole proton at 7.45 ppm were observed. Moreover, quartet and triplet of CH_2_ and CH_3_ were observed at 4.21 ppm and 1.23 ppm respectively.

### Antibacterial assay of synthesized compounds

All experiments were conducted in the boundaries of ethical principles and there was no involvement of human or animal samples in this project.

Selected clinical gram-negative and gram-positive bacteria were subjected to antibiotic disks diffusion by Kirby Bauer assay and their pattern of resistance were determined. The resistance pattern of synthesized derivatives is given in Table 2.

**Table 2:** Antibiotic resistance profiling

Antibiotics	Zone of inhibition (mm)						
	Gram Negative bacteria		Gram Positive bacteria		CLSI guideline		
	*Escherichia coli*	*Pseudomonas aeruginosa*	*Staphylococcus epidermidis*	*Staphylococcus aureus*	S	I	R
Cefepime	9	15	–	–	≥ 18	15–17	≤ 14
Ciprofloxacin	No zone	20	20	22	≥ 21	16–20	≤ 15
Imipenem	20	18	–	–	≥ 23	20–22	≤ 19
Cefoxitin	17	No zone	14	15	≥ 18	15–17	≤ 14
Ampicillin	No zone	No zone	18	No zone	≥ 17	14–16	≤ 13
Aztreonam	12	17	No zone	No zone	≥ 21	18–20	≤ 17
Tetracycline	8	No zone	No zone	18	≥ 15	12–14	≤ 11
Ceftazidime	10	14	–	–	≥ 21	18–20	≤ 17
Minocyclin	13	13	–	18	≥ 19		≤ 14
Gentamycin	No zone	18	16	18	≥ 15	13–14	≤ 12
Co-trimoxazole	No zone	12	No zone	15	≥ 16	11–15	≤ 10
Colistin	9	12	No zone	No zone	≥ 11		≤ 10
Clindamycin	No zone	No zone	No zone	No zone	≥ 21	15–20	≤ 14
Vancomycin	No zone	No zone	No zone	15	≥ 22		≤ 21
Doxicyclin	12	12	–	18	≥ 16		≤ 12
Erythromycin	7	No zone	No zone	20	≥ 23	14–22	≤ 13
Chloramphenicol	–	–	23	20	≥ 32	16	≤ 8

Susceptible (S), intermediate (I) and resistant (R)

### Quantification

**Fig. 2:**
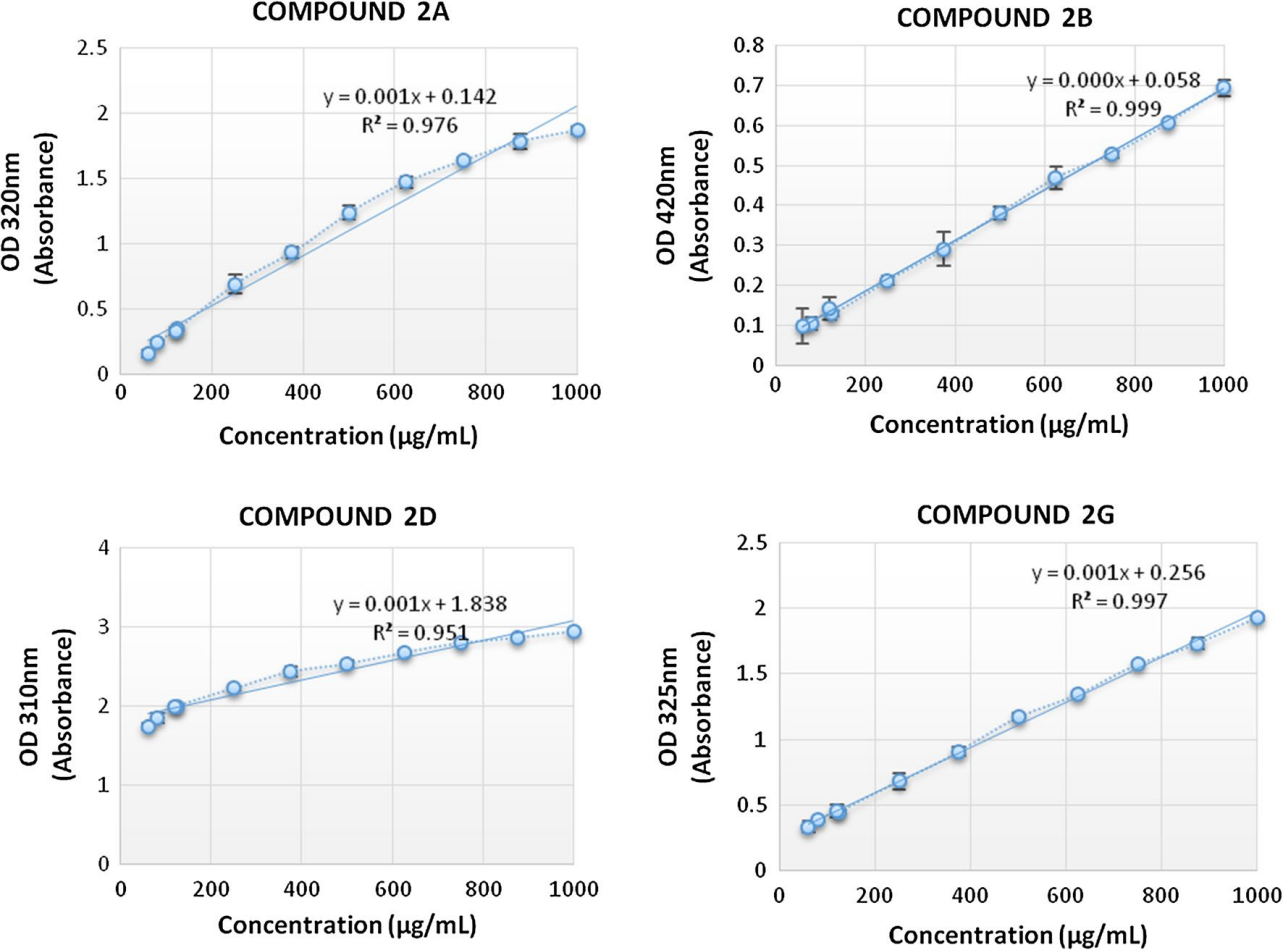
Optical Density (OD). Figure 1 illustrates the quantification of compounds by UV visible spectrophotometer. Linear line indicates the fitness of data against regression line

Spectrophotometric analysis was utilized for the quantification of compounds 2a, 2b, 2d and 2g. After performing wave scan the values obtained for λ_max_ were 2a at 320 nm, 2b at 420 nm, 2d at 310 nm and 2g at 325 nm. λ_max_ values were used to measure dilutions optical density (OD). These OD values were used to plot the standard curve and coefficient of determination (R^2^) value was calculated. The curve was plotted between OD values on y-axis while the concentration values on horizontal x-axis. From regression analysis, the R^2^ values obtained for compound 2a, 2b, 2d and 2g were 0.976, 0.999, 0.951 and 0.997 respectively. Values closer to 1 indicated the fitness of the data against the regression line. The graphs plotted are given in Fig. 2.

### Minimum inhibitory concentration (MIC)

All the synthesized compounds manifested mild to moderate antibacterial potential. The lowest concentration of the drug exhibiting bacteriostatic action is stated to be the Minimum inhibitory concentration (MIC) of the drug. The MIC of synthesized derivatives is given in Table 3. Compound 2a and 2b showed quite good MIC of 250 µg/mL against gram-positive *Staphylococcus epidermidis* (MDR). Likewise compound 2d and 2g exhibited MIC of 250 µg/mL against gram-positive bacteria *S. aureus* (MDR). Compounds 2d and 2g exhibited MIC of 375 µg/mL against *E. coli* (gram-negative) (MDR). While compounds 2a and 2b showed MIC of 375 µg/mL against *P. aeruginosa* (gram-negative) (MDR).

**Table 3:** MIC of synthesized compounds

Bacterial strains	Minimum inhibitory concentration (MIC) µg/mL									
	40	60	125	250	375	500	625	750	875	1000
*Escherichia coli*	–	–	–	–	2d, 2g	2a, 2b	–	–	–	–
*Pseudomonas aeruginosa*	–	–	–	–	2a, 2b	2d, 2g	–	–	–	–
*Staphylococcus epidermidis*	–	–	–	2a, 2b	2d, 2g	–	–	–	–	–
*Staphylococcus aureus*	–	–	–	2d, 2g	2a, 2b	–	–	–	–	–

### Antifungal activity

Compounds 2a, 2b and 2d showed maximam antifungal potential against *Candida glabrata* (ATCC 62934) with a zone of inhibition (mm) of 13.0, 21.0 and 13.1 respectively. *Candida albicans* (ATCC 60387) showed sensitivity to compounds 2a, 2b 2d, 2f and 2g with a zone of inhibition (mm) of 20.0, 13.8, 19.1, 15.4 and 14.9 respectively **(**Table 4).

**Table 4:** Antifungal activity of synthesized compounds (**1a**, **2a**–**2g**)

Compounds	Antimicrobial activity of synthesized compounds (zone of inhibition mm)	
	*Candida glabrata*	*Candida albicans*
**1a**	11.0	11.1
**2a**	13.0	20.0
**2b**	21.0	13.8
**2c**	10.9	11.0
**2d**	13.1	11.8
**2e**	11.5	19.1
**2f**	10.8	15.4
**2g**	12.0	14.9
**Nystatin**	19.1	19.3

Concentration of each compound (0.5 mg/50 μL)

### Insilico studies

Best compounds are selected computationally by analyzing them for their chemo-informatics and ADMET properties as given in Tables 5 and 6. According to literature, established qualifying range for log P value is (− 0.4 to 5.6). All compounds log p value lie within the limits. Molar refractivity should lie between (40 to 130) while molecular weight limits are (160 to 480). Moreover, the required number of atoms of compounds should lie between (20 to 70). Number of rotatable bonds should be less than 10. All compounds have 4 rotatable bonds except the compounds 2e and 2h that are having 5 rotatable bonds. All the compounds are fulfilling the set criteria for above parameters. Moreover, the drug likeliness following the Lipinski rule is also fulfilled. That’s why these compounds can be used as drug candidates.

**Table 5:** Chemo-informatics of compounds **2a**–**2h**

Chemo-informatics	2a	2b	2c	2d	2e	2f	2g	2h
Mol. formula	C_13_H_12_N_2_O_2_S	C_13_H_12_N_2_O_3_S	C_13_H_12_N_2_O_3_S	C_13_H_12_N_2_O_3_S	C_14_H_14_N_2_O_4_S	C_14_H_14_N_2_O_2_S	C_14_H_14_N_2_O_3_S	C_15_H_17_N_3_O_2_S
Molecular weight (g/mol)	260.31	276.31	276.31	276.31	306.33	274.33	290.33	303.38
No. of HBA	5	6	6	6	7	5	6	6
No. of HBD	0	1	1	1	1	0	1	0
No. of rotatable bonds	4	4	4	4	5	4	4	5
Mol. Log*P*	3.36	2.98	3.10	3.10	3.07	3.33	2.95	3.48
Mol. PSA (Å^2^)	39.17	55.71	56.78	56.78	63.34	38.50	55.04	41.97
Molar refractivity (cm^3^)	73.33	74.18	74.18	74.18	79.99	77.75	78.60	86.13
Density (g/cm^3^)	1.22	1.32	1.32	1.32	1.32	1.20	1.29	1.19
Surface tension (dyne/cm)	46.5	51.0	51.0	51.0	48.5	43.8	47.8	43.4
Polarizability (cm^3^)	29.07	29.40	29.40	29.40	31.71	30.82	31.16	34.14
Molar volume (Å^3^)	238.45	248.98	249.07	249.00	281.72	263.23	274.41	288.00
Drug likeness	− 0.32	− 0.19	0.30	0.22	0.20	− 0.46	0.15	− 0.48
Lipinski’s rule validation	Yes	Yes	Yes	Yes	Yes	Yes	Yes	No

*HBA* hydrogen bond donor, *HBD* hydrogen bond acceptor, *PSA* polar surface area

**Table 6:** ADME assessment of compounds **2a**–**2h**

ADME properties	2a	2b	2c	2d	2e	2f	2g	2h
Absorption								
WS (log mol/L)	− 3.147	− 3.599	− 3.644	− 3.8	− 3.901	− 3.538	− 3.742	− 4.122
IS (% abs)	95.067	92.136	93.112	92.486	94.048	94.329	91.609	95.929
SP (logKp)	− 2.46	− 2.865	− 2.826	− 2.837	− 2.943	− 2.508	− 2.908	− 2.61
Distribution								
BBBP (logBB)	0.055	− 0.545	− 0.549	− 0.574	− 0.726	0.389	− 0.518	0.291
CNSP (logPS)	− 2.215	− 2.407	− 2.905	− 2.915	− 2.973	− 2.768	− 2.864	− 2.772
Metabolism								
CYP3A4 inhibitor	Yes	No	No	No	No	No	No	No
Excretion								
TC (log mL/min/kg)	0.297	0.248	0.192	0.07	0.273	0.254	0.278	0.278

*WS* water solubility, *IS* intestinal solubility (% abs absorption), *SP* skin permeability, *BBBP* blood brain barrier permeability, *CNSP* Central Nervous System permeability, *TC* total clearance

Toxicity profile was determined by using TOXTREE and pkCSM online tools. All the compounds employed were having a therapeutically safe profile excluding compound 2h, which holds genotoxic potential. Therefore, compound 2h was not utilized in the docking step. Table 7 contains the toxicity profile of designed compounds.

**Table 7:** Toxicity Profile of compounds **2a**–**2h**

Toxicity	2a	2b	2c	2d	2e	2f	2g	2h
Nongenotoxic carcinogenicity	No	No	No	No	No	No	No	No
Genotoxic carcinogenicity	No	No	No	No	No	No	No	Yes
Invitro mutagenicity alerts (Ames test)	No	No	No	No	No	No	No	No
Potential for *S. typhimurium*	No	No	No	No	No	No	No	No
Max. tolerated dose log (mg/kg/day)	0.295	0.387	0.684	0.711	1.099	0.758	0.393	0.306
ORAT (LD50) (mol/kg)	2.343	2.285	2.427	2.343	2.599	2.519	2.366	2.571
HT	Yes	No	No	No	No	Yes	No	Yes
SS	No	No	No	No	No	No	No	No

*HT* hepatotoxicity, *SS* skin sensitization, *ORAT* oral rat acute toxicity

**Fig. 3:**
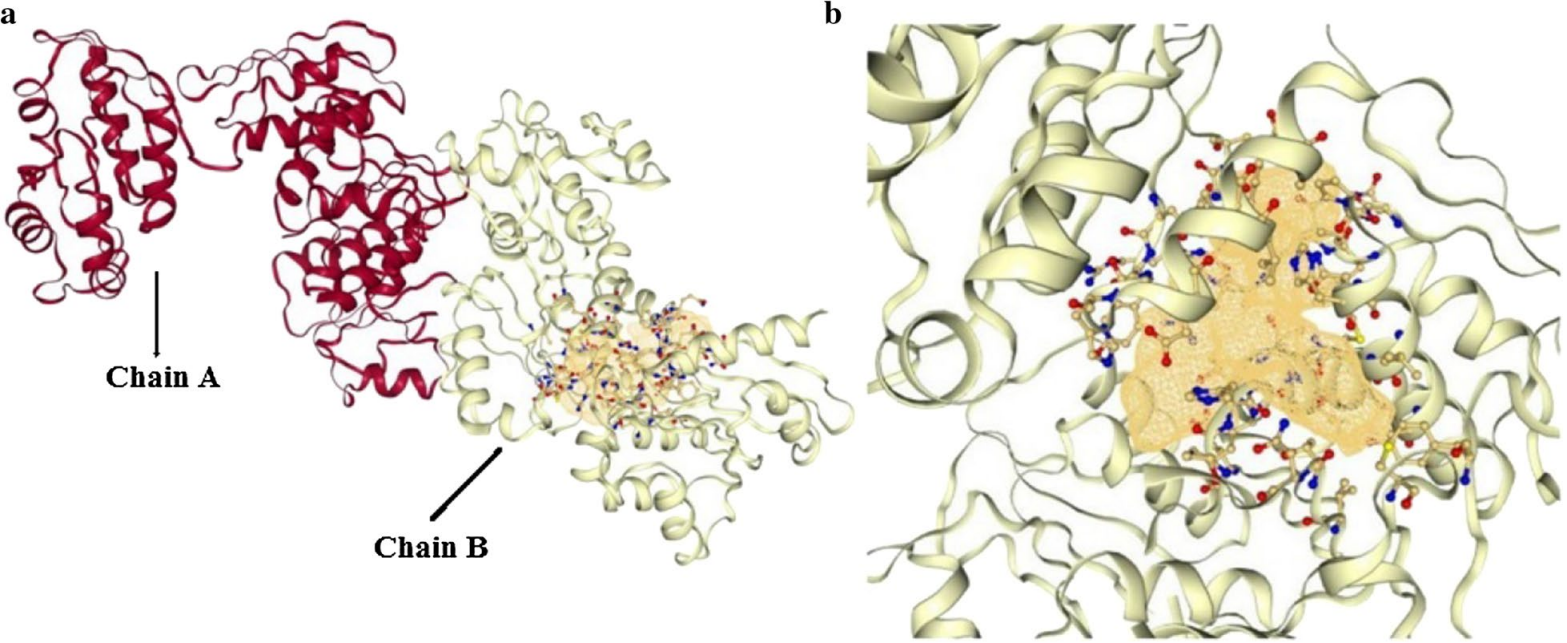
**a** depicts the ligand binding pocket of UDP-*N*-acetylmuramate/l-alanine ligase target protein while **b** is showing the enlarged view of chain B ligand binding pocket

Our designed compounds were docked with the target enzyme UDP-*N*-acetylmuramate/l-alanine ligase and binding affinities were determined. The binding pocket of target enzyme was identified through Dogsitescorer. All the compounds docked with the biggest pocket of the enzyme target located on chain B. The volume and surface of the pocket is 963.07 [Å^3^] and 1100.44 [Å^2^] respectively. While the pocket possesses quite good drug score of 0.77. Figure 3 illustrates the druggable pocket of UDP-*N*-acetylmuramate/l-alanine ligase target enzyme.

**Fig. 4:**
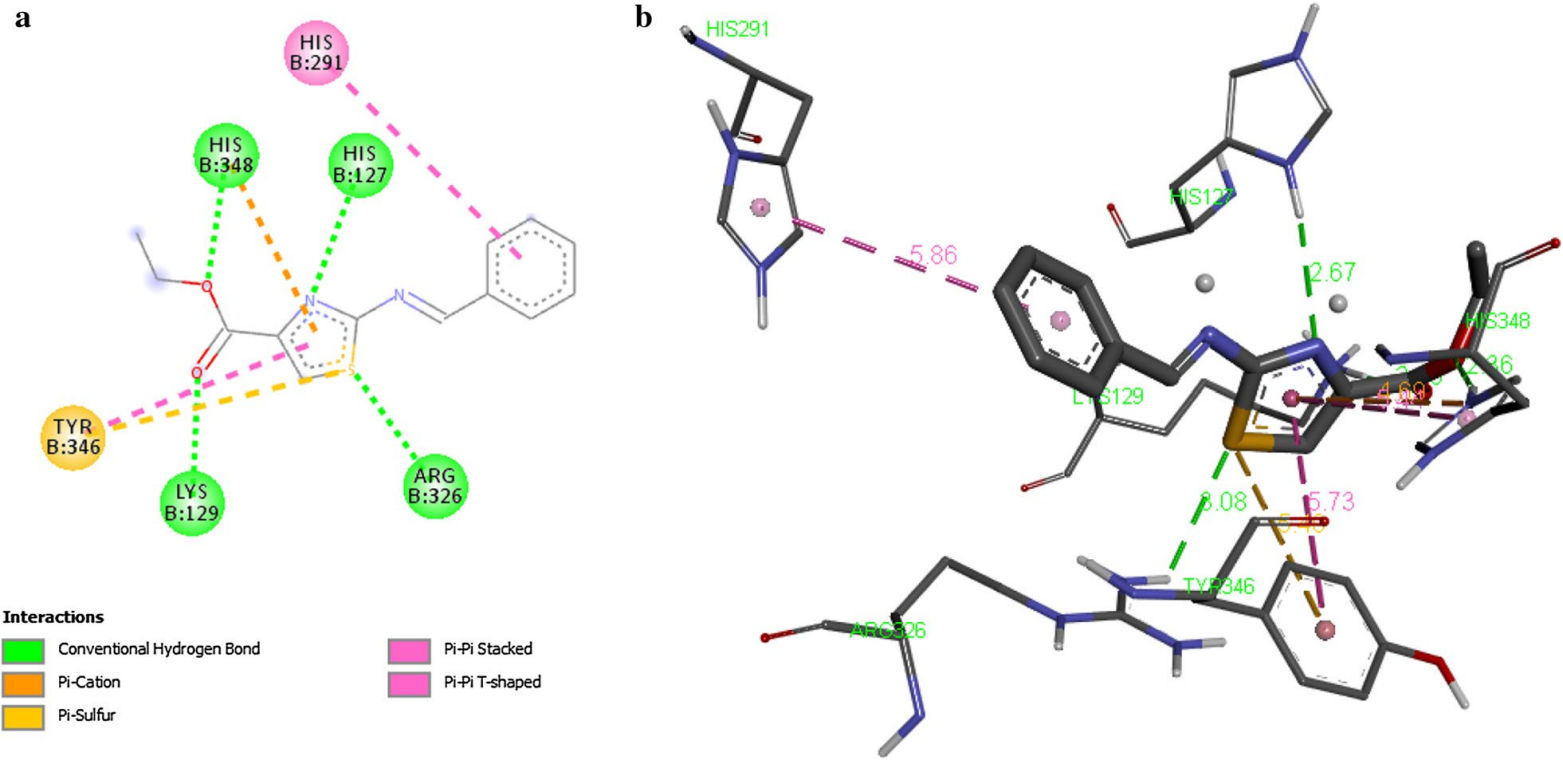
Interaction of UDP-*N*-acetylmuramate/l-alanine ligase with compound 2a. **a** Two dimensional diagram of protein–ligand interaction of UDP-*N*-acetylmuramate/l-alanine ligase with compound 2a. **b** Three dimensional view of compound 2a docking with UDP-*N*-acetylmuramate/l-alanine ligase, illustrates the pocket amino acids and bond distances of interacting groups of protein receptor and the ligand

**Fig. 5:**
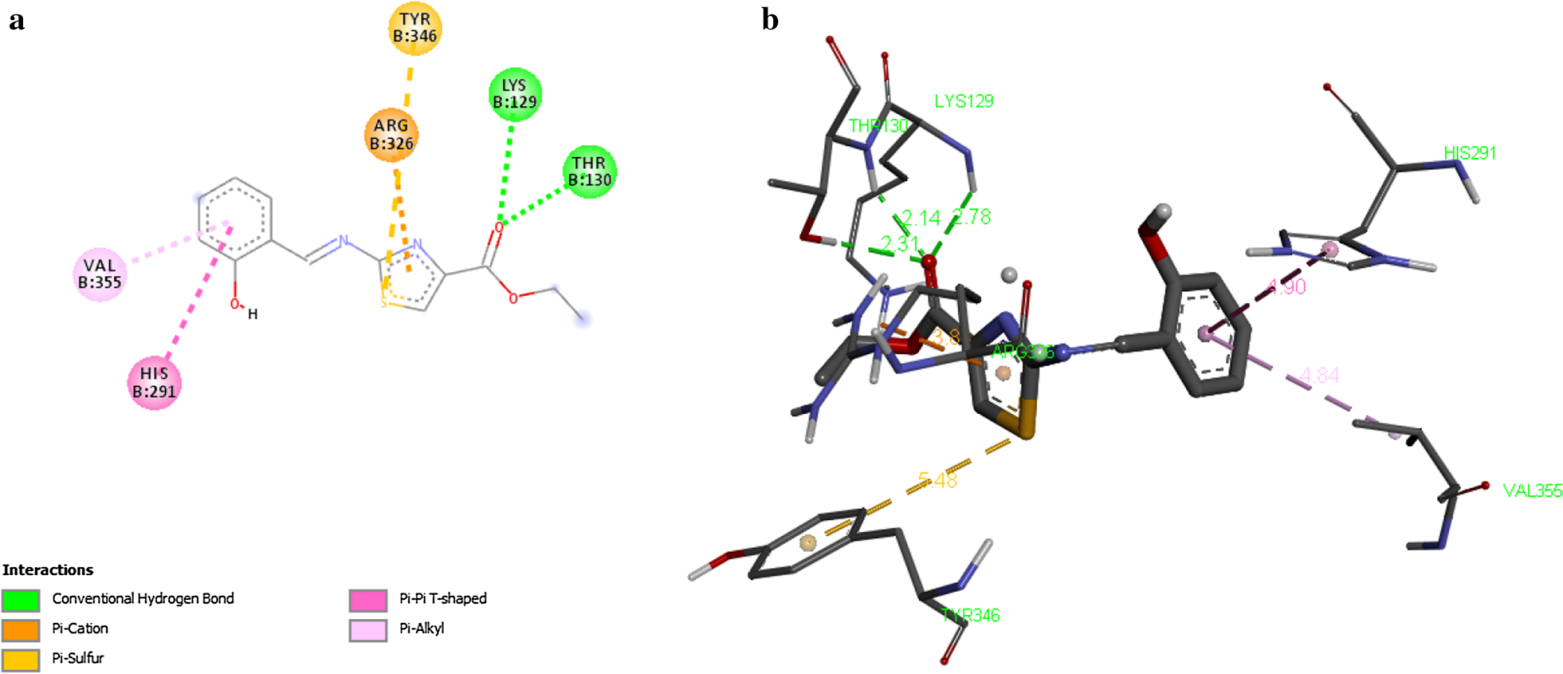
Interaction of UDP-*N*-acetylmuramate/l-alanine ligase with compound 2b. a Two dimensional diagram of protein–ligand interaction of UDP-*N*-acetylmuramate/l-alanine ligase with compound 2b. **b** Three dimensional view of compound 2b docking with UDP-*N*-acetylmuramate/l-alanine ligase, illustrates the pocket amino acids and bond distances of interacting groups of protein receptor and the ligand

**Fig. 6:**
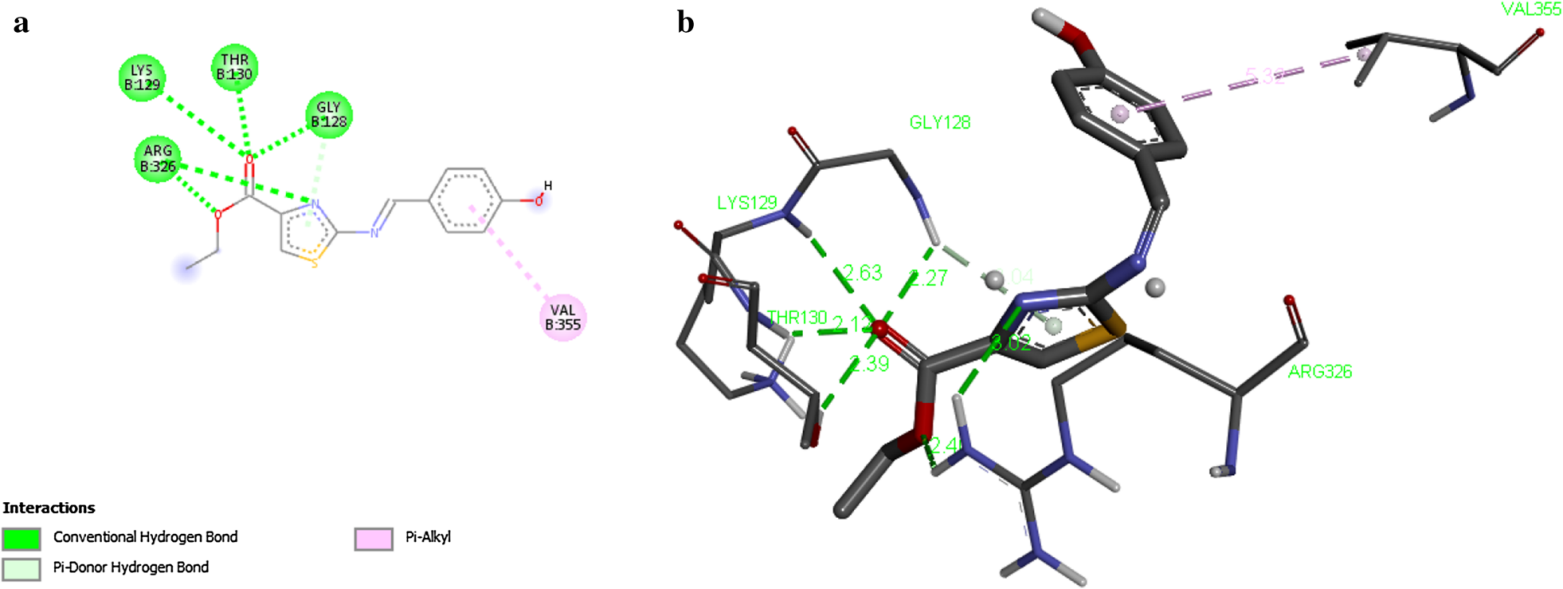
Interaction of UDP-*N*-acetylmuramate/l-alanine ligase with compound 2d. **a** Two dimensional diagram of protein–ligand interaction of UDP-*N*-acetylmuramate/l-alanine ligase with compound 2d. **b** Three dimensional view of compound 2d docking with UDP-*N*-acetylmuramate/l-alanine ligase, illustrates the pocket amino acids and bond distances of interacting groups of protein receptor and the ligand

**Fig. 7:**
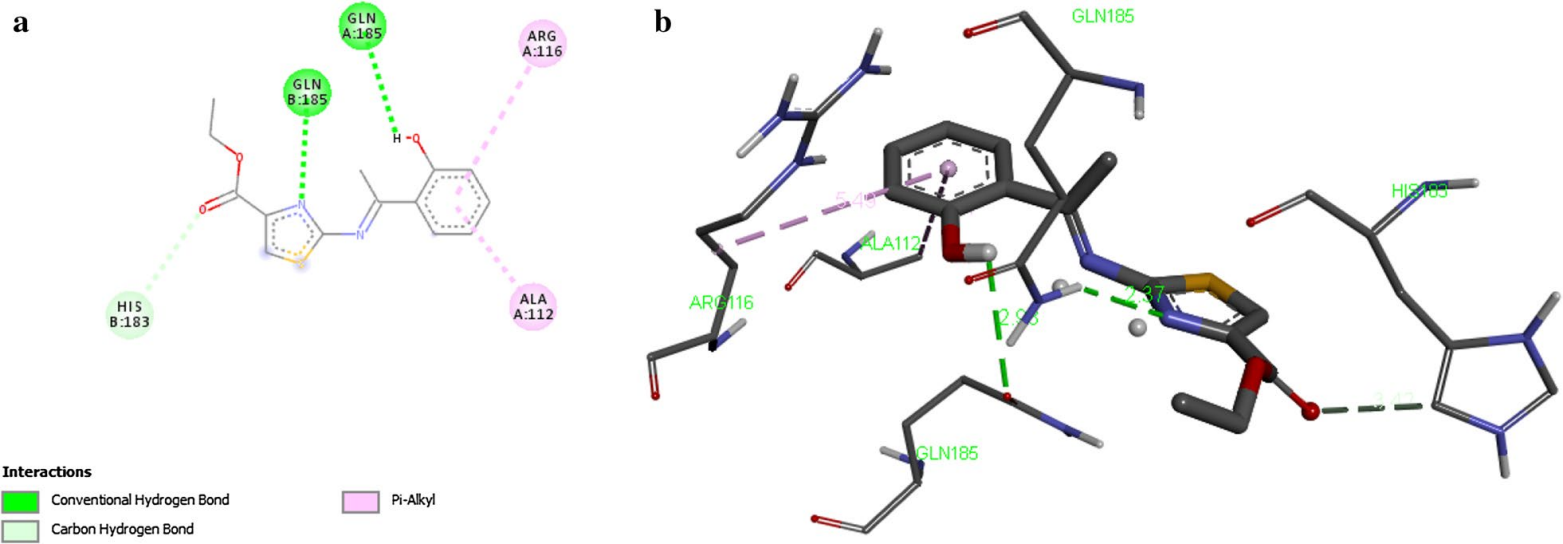
Interaction of UDP-*N*-acetylmuramate/l-alanine ligase with compound 2g. **a** two dimensional diagram of protein–ligand interaction of UDP-*N*-acetylmuramate/l-alanine ligase with compound 2g. **b** Three dimensional view of compound 2g docking with UDP-*N*-acetylmuramate/l-alanine ligase, illustrates the pocket amino acids and bond distances of interacting groups of protein receptor and the ligand

Results of docking of compounds are displayed (Table 8) in terms of free binding energy of ligand also stated as binding affinity (kcal/mol), root mean square deviation (RMSD) upper bound along with values of RMSD lower bound of all the docked ligands as well. Strength of binding interactions between ligand and receptor is termed as affinity. Calculation of RMSD values is based on the best binding pose and it employs movable heavy atoms only. Among the compounds 2a, 2b, 2d and 2g; 2b shown the strongest binding affinity of − 7.6 kcal/mol against UDP-*N*-acetylmuramate/l-alanine ligase. While compounds 2a, 2d and 2g exhibited binding affinity of − 6.8, − 7.3, − 7.1 and − 6.9 kcal/mol respectively. It is inferred from the binding affinities that the compounds having hydroxyl gyoup substituted on benzene ring possess strong binding affinity as compared to others. These designed compounds could be considered as antagonist lead molecules for target UDP-*N*-acetylmuramate/l-alanine ligase. The enzyme-ligand binding interactions of compounds 2b, 2c, 2d, and 2g with target UDP-*N*-acetylmuramate/l-alanine ligase are shown in Figs. 4, 5, 6, 7 respectively.

**Table 8:** Docking Scores of first three best docked posses of Compound **2a**, **2b**, **2d** and **2g** with UDP-*N*-acetylmuramate/l-alanine ligase

Target protein	Compound	Ligand modes	Binding affinity	rmsd/ub	rmsd/lb
UDP-*N*-acetylmuramate/l-alanine ligase	2a	0	− 6.8	0	0
	2a	1	− 6.7	28.075	26.052
	2a	2	− 6.6	28.511	26.881
	2b	0	− 7.6	0	0
	2b	1	− 7.0	28.764	27.311
	2b	2	− 7.0	28.373	27.402
	2d	0	− 7.3	0	0
	2d	1	− 7.0	28.595	27.20
	2d	2	− 7.0	28.136	26.504
	2g	0	− 7.1	0	0
	2g	1	− 6.9	7.342	2.41
	2g	2	− 6.8	3.16	2.568

*UDP* uridine diphosphate

## Conclusion

The aim of the study is to design and synthesize novel Schiff base derivatives of ethyl-2-aminothiazole-4-carboxylate. Characterization was done by FTIR, ^1^H NMR and ^13^C NMR spectral data and quantification is done through Multiskan™ GO Microplate Spectrophotometer. Synthesized derivatives are screened for their antibacterial potential against Multi drug resistant (MDR) clinical isolates. Moreover, synthesized derivatives also exhibited good antifungal activity against ATCC fungal strains. It is anticipated that these synthesized compounds are promising potent antibacterial therapeutic agents. In future it is aimed to develop a pharmacophore model from these compounds to have a best lead molecule for the UDP-*N*-acetylmuramate/l-alanine ligase target enzyme.

## Data Availability

All the relevant data supporting the conclusions of this article is included in the article.

## Abbreviations

ATCC: American Type Culture Collection
CDCl_3_: deuterated chloroform
DMSO: dimethyl sulfoxide
FTIR: Fourier transform infrared
MIC: minimum inhibitory concentration
NMR: nuclear magnetic resonance
SDA: sabouraud dextrose agar
TLC: thin layer chromatography
